# MetMap Enables Genome-Scale Methyltyping for Determining Methylation States in Populations

**DOI:** 10.1371/journal.pcbi.1000888

**Published:** 2010-08-19

**Authors:** Meromit Singer, Dario Boffelli, Joseph Dhahbi, Alexander Schönhuth, Gary P. Schroth, David I. K. Martin, Lior Pachter

**Affiliations:** 1 Computer Science Division, University of California at Berkeley, Berkeley, California, United States of America; 2 Children's Hospital Oakland Research Institute, Oakland, California, United States of America; 3 Department of Mathematics, University of California at Berkeley, Berkeley, California, United States of America; 4 Department of Molecular and Cell Biology, University of California at Berkeley, Berkeley, California, United States of America; 5 Illumina, Hayward, California, United States of America; University of British Columbia, Canada

## Abstract

The ability to assay genome-scale methylation patterns using high-throughput sequencing makes it possible to carry out association studies to determine the relationship between epigenetic variation and phenotype. While bisulfite sequencing can determine a methylome at high resolution, cost inhibits its use in comparative and population studies. MethylSeq, based on sequencing of fragment ends produced by a methylation-sensitive restriction enzyme, is a method for methyltyping (survey of methylation states) and is a site-specific and cost-effective alternative to whole-genome bisulfite sequencing. Despite its advantages, the use of MethylSeq has been restricted by biases in MethylSeq data that complicate the determination of methyltypes. Here we introduce a statistical method, MetMap, that produces corrected site-specific methylation states from MethylSeq experiments and annotates unmethylated islands across the genome. MetMap integrates genome sequence information with experimental data, in a statistically sound and cohesive Bayesian Network. It infers the extent of methylation at individual CGs and across regions, and serves as a framework for comparative methylation analysis within and among species. We validated MetMap's inferences with direct bisulfite sequencing, showing that the methylation status of sites and islands is accurately inferred. We used MetMap to analyze MethylSeq data from four human neutrophil samples, identifying novel, highly unmethylated islands that are invisible to sequence-based annotation strategies. The combination of MethylSeq and MetMap is a powerful and cost-effective tool for determining genome-scale methyltypes suitable for comparative and association studies.

## Author Summary

In the vertebrates, methylation of cytosine residues in DNA regulates gene activity in concert with proteins that associate with DNA. Large-scale genomewide comparative studies that seek to link specific methylation patterns to disease will require hundreds or thousands of samples, and thus economical methods that assay genomewide methylation. One such method is MethylSeq, which samples cytosine methylation at site-specific resolution by high-throughput sequencing of the ends of DNA fragments generated by methylation-sensitive restriction enzymes. MethylSeq's low cost and simplicity of implementation enable its use in large-scale comparative studies, but biases inherent to the method inhibit interpretation of the data it produces. Here we present MetMap, a statistical framework that first accounts for the biases in MethylSeq data and then generates an analysis of the data that is suitable for use in comparative studies. We show that MethylSeq and MetMap can be used together to determine methylation profiles across the genome, and to identify novel unmethylated regions that are likely to be involved in gene regulation. The ability to conduct comparative studies of sufficient scale at a reasonable cost promises to reveal new insights into the relationship between cytosine methylation and phenotype.

## Introduction

New methods that assay epigenetic modifications over the whole genome promise to reveal insights into cell differentiation and development [1]–[15]. Moreover, incorporation of genome-scale epigenetic data into case-control studies is now becoming feasible, and has the potential to be a powerful tool in the study of disease [16]. Recent evidence has suggested that epigenetic variation is heritable, and may underlie phenotypic variation in humans ([17], our own observation with the human and chimpanzee methylomes). Such comparative studies rely both on the ability to obtain genome-scale epigenomic information cheaply and efficiently, and on the availability of methods for analysis of the data produced.

Cytosine methylation, which in vertebrates is mostly confined to CG dinucleotides, cooperates with other epigenetic modifications to suppress transcription initiation [3], [18] (in this paper we denote a cytosine that is followed by a guanine as CG, rather than CpG, and similarly CCGG is equivalent to CpCpGpG. We leave the notation for CpG islands unchanged). In vertebrates, most CGs are methylated. However, early experiments with the methylation-sensitive restriction enzyme HpaII showed that unmethylated CGs are clustered in “HpaII Tiny Fragment Islands” [19]. These unmethylated islands are frequently active promoter elements. Methods used to annotate them on a genomic scale have been based only on sequence composition, because until recently genome-scale assessment of HpaII fragments has not been practicable. The methylation status of these regions, known as CpG islands, has not been considered in their annotation and is generally unknown. Genome-scale survey of the methylation status of CGs would enable the annotation of CpG islands based on their methylation states, rather than their sequence. Patterns of unmethylated islands differ among tissues, and changes in the methylation states of certain regions are associated with disease, particularly cancer [2], [3],[20]–[22].

High-throughput sequencing technologies have catalyzed the development of new methods for measuring DNA methylation. These methods can be broadly classified as *methyltyping* versus *methylome sequencing*, in analogy with *genotyping* versus *genome sequencing* for DNA. Methyltyping technologies allow for the assessment of genome-scale methylation patterns, while emphasizing low cost at the expense of high resolution. Assays based on sequencing avoid problems associated with hybridization to arrays. Examples include MethylSeq, which is based on digestion with a methylation-sensitive enzyme and is the focus of this paper, and RRBS which is based on digestion with a methylation-insensitive enzyme followed by bisulfite sequencing [3], [23]. In contrast to methyltyping, whole-genome bisulfite sequencing offers the ability to measure absolute levels of DNA methylation at single-nucleotide resolution [7], [24], [25], but it is expensive because it requires sequencing of whole genomes. The issues of cost and coverage are complicated by a number of other issues. In the case of bisulfite sequencing, conversion may not always be complete. Also, the analysis requirements for the different assays vary in difficulty. For these reasons, there has been a proliferation of methods whose pros and cons are constantly changing as sequencing technologies change. A recent analysis (Table 2 in [26]) suggested that MethylSeq is the method with the most favorable profile of pros and cons, with respect to the measures chosen for comparison. Table 1 summarizes characteristics of MethylSeq and of the most commonly used alternative methods. MethylSeq retrieves information spanning more of the genome than RRBS, because of a more favorable profile of fragment sizes produced by HpaII relative to MspI (see the discussion of size selection bias below and the Methods section).

**Table 1:** Features of different methyltyping techniques.

	Site specific	Pre-chosen regions	Spanning of human genome	Spanning of CpG islands	#CG sites	Bisulfite conversion	Read length	Constraints on analysis	Comparable with low amounts of input DNA
MethylSeq	Yes	No	9.2%	92.9%	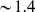 M	Not Needed	32bp suffice	Inference Procedure Needed	Yes
RRBS	Yes	No	8.1%	69.8%	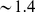 M	Needed	Longer = more coverage	Low Sequence Complexity	Yes
Affinity-based (MeDIP, mDIP, mCIP)-Seq	No	No	whole genome	all	-	Not Needed	32bp suffice	Binding Biases	No
Affinity-based (MeDIP, mDIP, mCIP)-Array	No	Yes	pre-chosen	pre-chosen	-	Not Needed	-	Binding Biases+Array Biases	No

For definition of spanning and determination of number of sites, see Methods. Constraints on analysis: for MethylSeq, see this paper. For RRBS, bisulphite conversion lowers sequence complexity, complicating alignments. Affinity based methods are complicated by effects of methylation density on binding, and by noise created by non-specific binding. Array methods are complicated by noise in the hybridization step. This table makes use of information from Laird 2010 [26].

**Figure 1:**
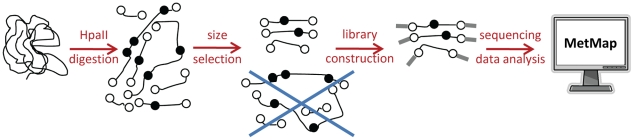
Determination of genome-scale methylation states with MethylSeq/MetMap. Genomic DNA is digested with the methylation-sensitive restriction enzyme HpaII. Unmethylated HpaII sites (open circles) are digested and thus found at the ends of restriction fragments, while methylated HpaII sites (black circles) are not digested. Restriction fragments are size-selected according to the Illumina protocol; fragments that are either too long or too short are removed. Fragments that pass the size-selection are used to construct sequencing libraries. After sequencing, the raw reads are aligned against the reference genome and processed with MetMap to derive maps of genome-scale methylation.

MethylSeq is a convenient methyltyping strategy because it is cost-effective, requires only small amounts of material, and avoids bisulfite conversion. Briefly, the assay works by digestion of DNA with a methylation-sensitive enzyme (HpaII) that cuts unmethylated CGs at CCGG sites. Subsequent sequencing and mapping to the genome reveals unmethylated CGs (Figure 1). Although the experiment is relatively simple, interpretation of the sequencing data is confounded by the dependence of read depth at a given site on the methylation status of neighboring sites. This has limited the use of MethylSeq; previous studies either pointed out the need for a method of site-specific normalization [1], or attempted to deal with the bias by removing problematic HpaII sites from the analysis [5](resulting in the loss from the analysis of more than 19% of HpaII sites in CpG islands, see Methods).

In order to make effective use of MethylSeq for genome-scale methyltyping we developed a freely available program, called MetMap, that infers methylation at individual CGs by modeling biases inherent in MethylSeq experiments. An additional important feature of MetMap is the annotation of strongly unmethylated islands (SUMIs) which, as opposed to the current definition of CpG islands, incorporate information from both a reference sequence and genome-scale methylation data. We have validated MetMap's site-specific analysis, as well as its unmethylated-island annotation, with bisulfite sequencing of specific sites.

We demonstrate the use of MethylSeq with MetMap by methyltyping four male human individuals, and annotating their unmethylated islands. We show that the picture revealed by such analysis is sufficient to survey methylation states across the genome. Such analysis gives significant insight into the methylome of each specimen, inside and outside of CpG islands, at site specific resolution. We show evidence that the mean extent of methylation of an island is more informative than the methylation state of the different sites in the island, because the correlation between the methylation states of any two samples improved when considering the mean. MetMap identifies numerous unmethylated regions, of varying lengths, which have not previously been annotated as CpG islands and are associated with other features indicative of transcriptional function. We conclude that MetMap leverages the cost-efficiency and practical ease of MethylSeq to produce informative genome-scale methylation annotations (methyltypes) that are suitable for both region- and site-specific comparative and case-control studies.

The remainder of this paper is organized as follows. We begin by explaining in detail significant biases present in MethylSeq experiments. We then describe the MetMap framework, which is designed to correct for such biases, starting with a description of MetMap's graphical model and continuing with a description of the software's different outputs. We then describe the validation of MetMap's procedure, using the methyltypes of four human individuals, and our discovery of new unmethylated regions in the human neutrophil genome, found through the use of MetMap on MethylSeq data. Finally, we discuss the advantages of using MetMap with MethylSeq to generate and analyze large numbers of samples, and outline our plans for the extension of MetMap's framework.

## Results

### Computational Model

#### Rationale

MetMap is a statistical inference framework that exploits MethylSeq data to accurately ascertain the extent of methylation across a genome. It uses a novel graphical model to assign probabilities of methylation at single-HpaII-site resolution, annotate regions of the genome that are hypomethylated along with a score indicating their extent of hypomethylation, and indicate the sites that are in the scope of the MethylSeq experiment and may be included in comparative studies.

**Figure 2:**
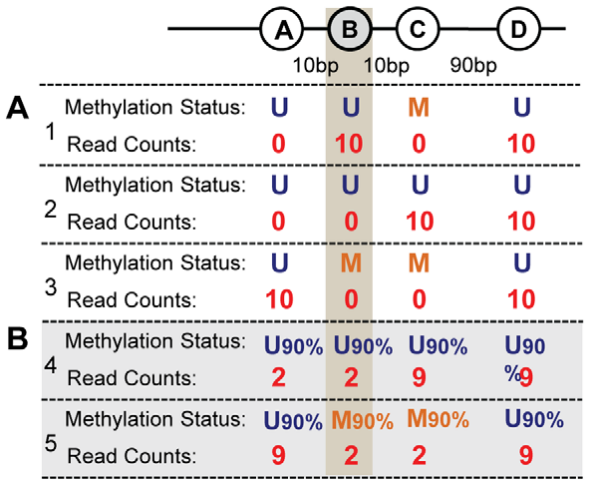
The methylation state of restriction site B cannot be determined by its read count alone. Suppose that due to the size selection step, only fragments of length 50–300bp are sequenced. The four adjacent restriction sites (denoted by circles) may have different methylation states, resulting in epialleles with different “neighborhood methylation structures” of B. Site B is sequenced only from fragments of type B–C–D, which are the product of alleles in which sites B and D are unmethylated (and cut) and site C is methylated (and not cut). (**a**) B is unmethylated in both case 1 and case 2, but it receives different read count values. In case 1 sites A,B and D are unmethylated and therefore digested by HpaII, giving fragments A–B of length 10bp and B–C–D of length 100bp. Fragment A–B is too short to be sequenced, but B–C–D has its ends sequenced. In case 2 all four HpaII sites are digested, giving fragments A–B, B–C and C–D. A–B and B–C are too short and are not sequenced, and so site B is not sequenced. In case 3, site B is methylated, is not cut by HpaII, and is not sequenced. Note that the read counts at site B alone cannot distinguish case 2 from case 3. (**b**) Analysis is complicated by heterogeneous methylation within a population of cells. The extent to which site B is methylated in the cell population cannot be determined given only the read count at site B. In case 4, although site B is cut in 90% of the cells, it is sequenced only infrequently, because site C is unmethylated and cut in 90% of the cells, resulting in a B–C fragment that is too short for sequencing. In contrast, in case 5 site B is cut in only 10% of the cells. But site C is methylated in 90% of cells, so the majority of the fragments in which site B has been cut will yield a B–C–D fragment and will be sequenced. Thus the methylation structure of neighboring restriction sites strongly influences the frequency with which a site will be sequenced.

A central feature of MetMap is its ability to normalize the bias introduced in short-read sequencing experiments in which the genome is not randomly fragmented. In MethylSeq experiments, all unmethylated HpaII sites are present at the ends of the digested fragments, but a size selection step required by the sequencing protocol limits sequencing to fragments of a narrow size range. The methylation status of the neighbors of an unmethylated HpaII site determines whether the fragments with this site at their ends will pass the size selection step and be sequenced (Figure 2.a). Moreover, the methylation state of CG sites can be heterogeneous even within a population of a single cell type [5], [14]. This complicates correction for the bias introduced by size selection (Figure 2.b). MetMap incorporates experimental data derived from a site's “neighborhood methylation structure” to achieve unbiased estimates of the probability of methylation, at single-site resolution.

Another bias is an issue of all “shotgun” sequencing experiments. The read count of a given fragment gives only an estimate of its abundance in the solution, and can be viewed as the number of times the fragment was randomly sampled. Therefore, different fragments present in the specimen in similar quantities will not always be sequenced to the same extent. The site-specific inference procedure used by MetMap considers the extent to which all fragments in a HpaII site's neighborhood were sequenced, so that more information is considered to determine the state of each HpaII site.

#### MetMap's graphical model

The analysis of a MethylSeq experiment by MetMap begins with the generation of a directed graphical model. The model's specific structure is determined by a reference genome and the specifications of the given experiment. We outline the different types of variables in MetMap's model, the dependencies between the variables, and how the data is incorporated into the “observed” states.

**Figure 3:**
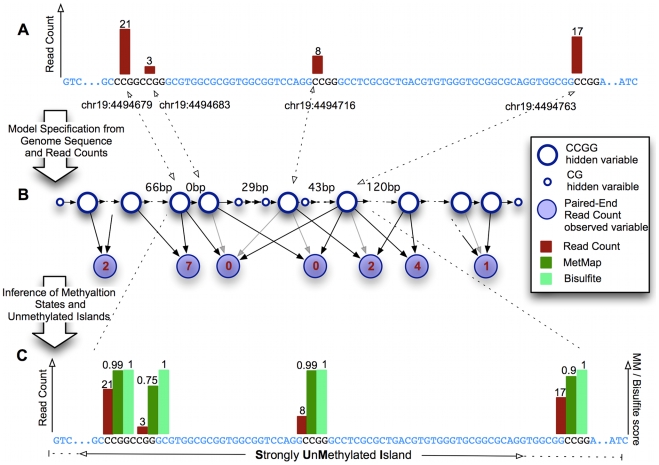
Inference of site-specific probabilities of unmethylation and annotation of strongly unmethylated islands from MethylSeq read counts. MetMap constructs a directed graphical model (b) from the genome and read counts (a). The methylation state of each CCGG site is represented by a random variable that also encodes whether it is in an unmethylated island. CpG sites are also represented in the model, with the distance between sites affecting the parameters. The read counts are used to set the state of the observed random variables corresponding to the possible sequenced fragments (for simplicity of representation, only a sample of these variables is outlined in the figure). The numbers in the blue circles represent normalized read counts. Dark edges correspond to boundaries of fragments. MetMap inferences of the extent of unmethylation (c) are shown alongside the values attained from a bisulfite sequencing validation. The raw read counts are scaled by the 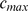 value chosen for sample 4 (Methods). Strongly Unmethylated Islands are annotated from the posterior distributions inferred at sites and the total read counts. The example shows part of an inferred SUMI on chromosome 19 from sample 4.

For a given reference genome, MetMap denotes every CG that is within a HpaII site (CCGG) by a random variable (denoted by 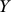
s, large open circles in Figure 3.b), and every non-HpaII CG by a random variable (denoted by 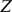
s, small open circles in Figure 3.b). Each HpaII site variable takes on one of six states. Each state incorporates one of the three values 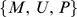 determining the site as methylated, unmethylated and heterogeneously methylated respectively, and one of the two values 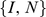 determining the site's presence (or not) in an unmethylated island. Each non-HpaII-site CG variable (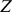) has two possible states: 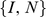. 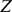 variables do not incorporate methylation values because there is no experimental data on non-HpaII CG sites. Every genomic fragment that could be produced by cleavage of any two HpaII sites, and which passes the size selection step, has a corresponding random variable (denoted by 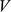
s, blue circles in Figure 3.b). In other words, these are all possible fragments in the given size range that have a HpaII site at each end. The 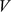 variables are the observed variables of the model. Each 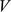 variable has a state determining the relative extent (0 being the lowest and 9 the highest) to which the specific fragment it represents was detected by MethylSeq. 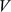 variables range over 0–9 to account for heterogeneous methylation of sites within a population of cells [5], [7] and are assigned by a normalization procedure that is applied to the read counts (Methods). In summary, the variables of the model have the following possible states:
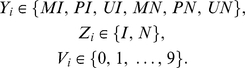



Dependencies between the variables are modeled using probability distributions of three types, making use of 54 parameters. The first type of probability distribution captures the dependencies between adjacent CG sites with respect to whether the sites are part of an unmethylated island, and is modeled by transition probabilities of a hidden Markov model (HMM) that incorporates the distances between adjacent sites. The second determines, for each 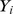, its probability of being in each of the three methylation values described above, given placement of the site in an unmethylated island (or not). The third type models the generation of MethylSeq experimental results, and thus the bias present in such experiments: the presence of a fragment (a 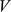 variable) in the data requires that it was cut at its ends, and not between them. MetMap incorporates values for 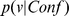, where 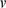 denotes a state of 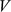, and 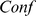 is some methylation value configuration of the 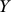 variables (ends and interior) on the fragment 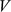 represents. Further details about the transition functions are found in the Methods section.

In summary, the reference genome specifies the structure of the graphical model, and the MethylSeq data is incorporated into this model by fixing the states of all of the 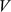 variables (Figure 3.a,b). This integrates into one inference model our knowledge of the MethylSeq data, of the main sources of experimental bias, and of the genome sequence.

#### Site-specific methylation probabilities

After building the probabilistic model and assigning the 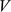 variables, MetMap infers, for each of the “hidden” 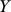 and 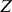 variables, the posterior probability over its states (Figure 3.c). To do this MetMap uses belief propagation on the junction-tree graph built from the directed model [27]. The computation is tractable and efficient because the tree width of the model is small (Methods). Given the posterior probabilities, the probability of each restriction site, 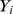, to be unmethylated or methylated is respectively:
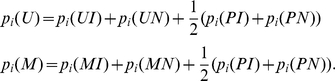



It is important to restrict analysis only to sites that are within the scope of the MethylSeq experiment, namely, to sites for which the MethylSeq experiment holds some information regarding their methylation state. MetMap identifies these sites from the structure of the graphical model (Methods) and outputs a file with coordinates of all CCGG sites in the scope of the experiment, along with their inferred 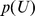 values.

#### Strongly unmethylated islands (SUMIs)

As unmethylated CGs tend to be clustered in vertebrate genomes, we would like to annotate the coordinates of these clusters. We call such regions SUMIs (strongly unmethylated islands) and emphasize that they are defined by experimental data and so are specific to a dataset. In MetMap's graphical model the posterior probability of a variable to be in an “unmethylated island” state is dependent on both the genome sequence and the experimental data (for any 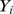 variable this posterior probability is 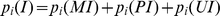). This could cause sites with a very high concentration of CGs around them to get high posterior probabilities of being in the “unmethylated island” state, regardless of experimental data at those regions. MetMap accounts for this by annotating SUMI regions based on several different properties: the inferred probabilities of being in an unmethylated island, of being unmethylated and the direct MethylSeq data (Methods). Each SUMI receives a score indicating the extent to which the entire SUMI is unmethylated. This score is the mean of the 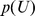 scores of all of the HpaII sites within that SUMI. SUMIs can be used as “comparative units”, facilitating the comparison of datasets. MetMap outputs a file with the coordinates and scores of the SUMIs inferred for a dataset.

### Evaluating MetMap's Performance

We carried out MethylSeq on specimens of a single homogeneous and uncultured cell type, the neutrophil, from four male humans. HpaII fragments were size selected in the range 50–300bp and sequenced on a first generation Illumina Genome Analyzer yielding 23,731,359 32bp reads. Although longer reads are currently available, reads for our assay only need to be sufficiently long so that they can be mapped correctly to the reference genome. The reads were aligned to the reference human genome (hg18 [28]) with Bowtie [29] resulting in 18,218,420 alignments (Table S1), and each of the four samples was analyzed with MetMap.

To infer methylation states from read depths, we first segmented the genome into 6,000 non-overlapping regions (of size 0.5Mbp) that could be analyzed separately. For each region, MetMap returned methylation probabilities for those CCGG sites for which information on site-specific methylation could be obtained from the MethylSeq experiment, and annotated SUMIs. The CCGG subset contained 59% of the CCGG sites (4.8% of all CG sites) in the human genome. Of the sites for which information could be obtained, 80% (1,035,243 sites) were outside CpG islands as annotated in the UCSC Genome Browser [30], and 20% (257,540 sites) were inside, resulting in a two-fold enrichment of the proportion of CCGG sites that are in such CpG islands.

To test whether MetMap was correcting bias in the raw counts (Figure 3), we directly determined the methylation status of 22 regions in the human genome using bisulfite sequencing [31] (Methods). Each CG in the bisulfite experiment received a score from the set (0,0.25,0.5,0.75,1) based on the observed proportion of alleles in which that site was unmethylated in a sample [32].

We correlated the bisulfite scores (taken as being the true methylation status) with the read counts and with the MetMap predictions. Each of the 46 validated sites had three different scores for the extent to which it was unmethylated: a bisulfite score, a read count score, and a MetMap score. The Pearson correlation coefficient between the raw read counts and the bisulfite values was 0.67 while the Pearson correlation coefficient between the MetMap methylation score of those sites and the bisulfite values was improved to 0.90.

As the bisulfite scores may be an imprecise measure of the true extent of methylation (Methods) we tested the sensitivity of our results to the bisulfite scores. We “adjusted” bisulfite scores, assigning to each value of the two sets of scores, the read-count set and the MetMap predictions set, a separate “adjusted” bisulfite value, that is within a predetermined range. The range available for adjustment was determined by the initial bisulfite score (Methods). After this adjustment, the correlation coefficient of the read counts with the bisulfite scores was 0.73 and the correlation coefficient of the MetMap scores with the bisulfite scores was 0.95. While the correlation values increased as expected, the difference between the performance of MetMap and that of read counts remains similar. This indicates that the improvement in using MetMap instead of raw read counts was not due to the procedure by which bisulfite scores were assigned.

Examples of MetMap's ability to accurately detect partially and fully methylated sites are shown in Figure 3, Figure S1 and Text S2. Both the extent and variability of methylation in a region are better predicted by MetMap than by the read counts.

**Figure 4:**
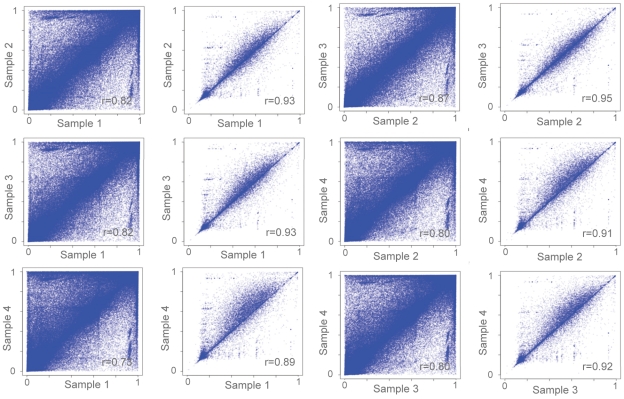
The average probability of methylation at SUMIs is highly stable across individuals of the same sex. All pairings among the four individuals tested are shown. On the left side of each pair the correlations between the site specific MetMap scores are presented for sites within SUMIs. On the right side of each pairing the correlations of the SUMI scores are presented. The distribution of the sites that are highly unmethylated in one sample but methylated to different extents in the other sample is discussed in Text S3.

To determine which parameter might be more informative for genome-scale methyltyping, we compared methylation states for individual sites and for SUMIs between pairs of samples. Although the methylation status of individual sites within SUMIs was variable, the average probability of methylation for the whole SUMI was consistent across individuals (Figure 4). This observation suggests that the mean methylation state of a SUMI is more constrained than the methylation states of the individual sites within it, and thus a change in mean SUMI methylation is more likely to have functional consequences than a change at a specific site. Based on this, we propose that the mean SUMI methylation status is the more informative parameter for comparative or association studies.

Similar read counts at orthologous restriction sites in two or more samples indicate that their methylation status is similar; however determination of their true extent of methylation requires a statistical method such as MetMap. Thus the degree of consistency observed among MetMap's site-specific inferences for different samples is supported by the high correlation of the corresponding raw read counts (e.g.: a correlation of 0.667 between sample 1 and sample 4).

### MetMap Identifies Novel Unmethylated Islands Associated with Promoters and Open Chromatin Regions

**Figure 5:**
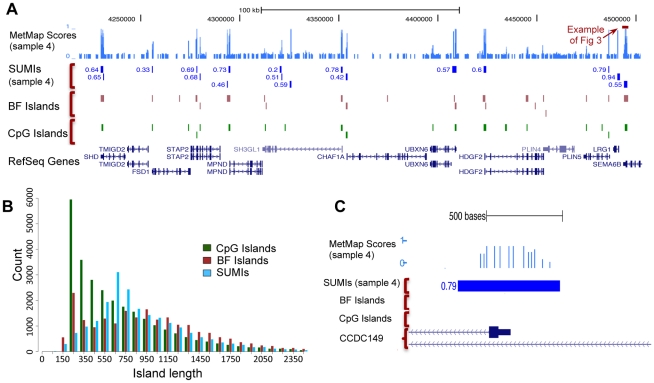
Strongly Unmethylated Islands (SUMIs) in the neutrophil methylome. Genomewide SUMI predictions (a) reveal strongly unmethylated islands that are proximal to genes and that do not always correspond to sequence-based annotations of CpG islands shown in the tracks ‘BF islands’ and ‘CpG islands’ (e.g., the promoter of LRG1 and in an intron of SH3GL1). (b) SUMI and BF island length distributions have a different shape than the CpG island length distribution, suggesting numerous short false positives in the latter. (c). Some SUMIs appear 5′ of alternative promoter sites.

We mapped the 20,986 SUMIs present in at least one of the four individuals, and examined their relationship to purely sequence based definitions of CpG islands (Figure 5.a). Of the 20,986 SUMIs present in at least one of the four individuals, 4,652 do not overlap UCSC CpG islands, and 7,055 do not overlap the “bona fide” islands [33] with an epigenetic score larger than 0.5 (as recommended by Bock et al. [33], termed here BF islands). This result is consistent with the higher specificity, but lower sensitivity, of BF compared to UCSC island prediction. Details regarding the extent of overlap between SUMIs and the BF and UCSC islands can be seen in Table 2.

**Table 2:** Counts of the SUMIs annotated in the four human neutrophil samples.

	All Neutrophil SUMIs	Overlapping CGIs	Not Overlapping CGIs	Overlapping BFIs	Not Overlapping BFIs	Not Overlapping CGIs or BFIs
Sample 1	16,903	14,071	2,832	12,076	4,827	2,266
Sample 2	17,595	15,008	2,587	12,834	4,761	2,044
Sample 3	18,178	15,273	2,905	13,082	5,096	2,308
Sample 4	18,699	15,274	3,425	13,229	5,470	2,729
Union	20,985	16,334	4,651	13,931	7,054	3,797
Intersection	14,308	12,838	1,470	11,123	3,185	1,116

Union - The set of regions annotated as a SUMI in at least one of the four individuals. Intersection - The set of regions annotated as a SUMI in all four individuals. CGIs - UCSC CpG islands. BFIs - BF-islands.

We compared the length distribution of our SUMIs with the length distributions of both the UCSC and BF islands (Figure 5.b). SUMIs were similar to BF islands, but the length distribution of the UCSC CpG islands resembled a geometric distribution. The process by which UCSC CpG islands are annotated will produce false positives that follow a geometric length distribution, with the number of false positive CpG islands increasing as a function of decreasing length (Methods). Since the length distributions of SUMIs and BF islands do not follow the same trend as the UCSC CpG island distribution, it is probable that at the shorter lengths the majority of predicted UCSC CpG islands are false positives. SUMIs did not overlap completely with BF islands: of the 21,626 BF islands, 13,899 were identified as SUMIs. BF islands are determined with a support vector machine that uses epigenetic data from multiple sources to train its prediction model. In contrast, MetMap's SUMI predictions originate from an experimental signal for unmethylation in the cell type analyzed. The probable explanation for the MetMap/BF discrepancy is that the two methods have used epigenetic data from different tissues. More data from distinct cell types will shed light on this issue.

We therefore validated with direct bisulfite sequencing five regions that are annotated as part of both a UCSC CpG island and a BF island, and did not overlap with SUMIs; we also sequenced three regions in BF islands that did not overlap with SUMIs or with UCSC CpG islands. In all cases those regions were validated as methylated in the neutrophil samples (Figure S1.j–q). This is consistent with the notion that while these islands might be unmethylated in other cell types, they are methylated in the neutrophil. We analyzed four cases of SUMIs with scores higher than 0.5 that overlapped UCSC CpG islands but not BF islands (Figure S1.a–d). In each SUMI a region was picked and bisulfite sequenced. All four regions were determined as fully unmethylated (all CG sites received a score of 1).

3,797 SUMIs do not overlap with BF islands or CpG islands, revealing new regions that are unmethylated in neutrophil cells. Of these novel SUMIs, 2,317 (61%) are within regions experimentally determined by the ENCODE project as open chromatin (Methods), 1,882 (50%) are within regions determined as conserved by the 17-way UCSC conservation track, 2,274 (60%) are within 2Kbp of RefSeq genes, and 837 (22%) are within 2Kbp of the 5′ end these genes (Figure. 5.c and Table 3).

**Table 3:** Percentages of neutrophil SUMIs, UCSC CpG islands and BF-islands that overlap regions associated with functionality.

	Human Neutrophil SUMIs	UCSC CpG islands	BF islands	Novel SUMIs
Open Chromatin	70.0%	52.9%	65.3%	61.0%
UCSC 17-way Conservation Track	71.1%	68.5%	76.2%	49.6%
Gene Regions	76.9%	77.7%	79.7%	59.9%
TSS Regions	59.8%	52.2%	61.4%	22.0%

“Open Chromatin” - the union of the regions determined by the ENCODE project as open chromatin in five different cell types (Methods). For the gene regions and TSS regions the RefSeq genes were used, and a window of 2Kbp was taken around each gene/TSS.

**Figure 6:**
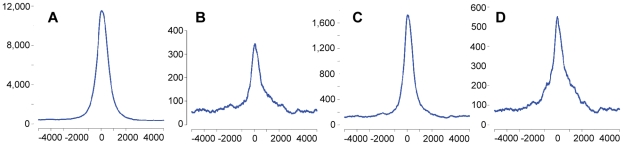
Transcription start sites and their close surroundings are enriched with novel SUMIs. The number of SUMIs that overlap each location within 5Kbp from RefSeq transcription start sites is shown for (a) all neutrophil SUMIs (b) Novel SUMIs (SUMIs that do not overlap UCSC CpG islands or BF islands) (c) SUMIs that do not overlap BF islands (d) SUMIs that do not overlap UCSC CpG islands.

Consistently with their similarity to conventional CpG islands, SUMIs are enriched near the transcription start sites (TSSs) of RefSeq genes, with a preference for the downstream side (Figure 6.a). We observe the same property also when we consider novel SUMIs alone (Figure 6.b), or when we consider only SUMIs that do not overlap UCSC CpG islands (Figure 6.c) or BF-islands (Fig. 6.d). This indicates that the distribution of novel SUMIs around the TSSs does not originate from a characteristic present in only one of these sets. We find that the proportion of SUMIs that maps at a distance from TSSs is larger for novel SUMIs than for all SUMIs, but that novel SUMIs have a degree of association with open chromatin similar to that observed for all SUMIs (Table 3); this suggests that novel SUMIs may often represent distal regulatory sequences.

## Discussion

The possibilities and potential of DNA methylation analysis with new sequencing technologies have been described as a “revolution” [26]. The vast number of methods for methylation analysis, along with many papers describing exciting findings, suggests that this revolution is underway. For the foreseeable future, methods that rely on the construction of a sequencing library produced by methyl-sensitive enzymes, followed by sequencing to measure methylation, are the practical approach for the analysis of large numbers of samples [26]. The efficient use of MethylSeq data requires a computational method that can infer true methylation states by considering biases inherent in the technical method. We have developed MetMap, which makes it possible to use MethylSeq for genome-scale methyltyping. MetMap facilitates the rapid calling of restriction-site-specific methylation, and of unmethylated regions, to produce methylation maps that are suitable for comparative analysis. Validation of MetMap calls with bisulfite sequencing shows that it compensates for bias present in the MethylSeq data. MetMap can combine experimental data and genome sequence to identify many strongly unmethylated islands (SUMIs) that were previously unannotated, suggesting that it can identify novel functional regions.

The annotation of experiment-specific strongly unmethylated islands (SUMIs) reconciles the original definition of CpG islands, based on their sensitivity to methylation-sensitive restriction enzymes [19] with the sequence-based definitions now used. The definition of SUMIs is functionally more exhaustive than the standard definition of CpG islands, since it couples sequence clues to methylation (abundance of CpGs) with experimental measurements of methylation. In our comparison of four humans, we noted that the average methylation states of SUMIs were more conserved among individuals than the methylation states of sites within them, suggesting that average methylation is more likely to be functionally important and so is a more informative parameter. SUMIs lie proximal to genes (77% are within 2Kbp of genes; 60% are within 2Kbp of the 5′ end), and are likely to be directly involved in regulation of gene expression.

Overall, we predicted 3,797 SUMIs that do not overlap UCSC CpG islands or BF islands. Their sequence conservation and correlation with open chromatin suggests that they are functional, but they are less frequently associated with transcription start sites than the general set of SUMIs. We speculate that many novel SUMIs are enhancers. The discovery of these novel regions illustrates the utility of using experimental data to annotate CpG islands.

As more methylation data becomes available, the MetMap program we have developed can be refined and improved. For example, with the advent of methylation-based case-control studies, it should be possible to define methyl-haplotypes and to leverage MetMap to explore variation within and between individuals. MetMap's graphical model can also be used to learn the dependencies between the methylation states of neighboring CG sites, which will expand the scope of MethylSeq experiments to include sites that are not directly assayed. As more data-types are produced together with methylation experiments, we envision expanding MetMap to include information from related genomes, and possibly other related measurements. Ultimately, we look forward to the coupling of methylation data with other functional information, including expression measurements and chromatin structure assays, to fully reveal the roles and consequence of DNA methylation.

## Methods

### Ethics Statement

Human samples were collected with CHORI's IRB approval after obtaining informed consent.

### Software

The MetMap software takes as input: (1) the mapped reads of a MethylSeq experiment, (2) the boundaries on the lengths of the fragments sequenced (determined by the size-selection step), and (3) a reference genome. It outputs two files: (1) a list of the HpaII sites in the scope of the experiment with their MetMap scores, and (2) a list of SUMI regions with their scores.

MetMap is free, open source software, and can be downloaded from the following site:

http://www.cs.berkeley.edu/meromit/MetMap.html

### Evaluating Per-Site Coverage and Span of Methods for Methyltyping

In the Methylseq experiment, information regarding the methylation state of a CCGG site can be obtained for the subset of CCGGs that are present on some fragment that has CCGG sites at its ends and that passes the size selection step (see “CG sites in the scope of the MethylSeq experiment” section for details). We computed the number of CCGGs of the human genome that fulfill this criterion to be 1,349,378.

In the RRBS protocol the genome is digested with the methylation-insensitive restriction enzyme MSPI (which cuts at CCGG sites), and the fragments of size 40–220bp are size-selected and have their ends sequenced (after bisulfite treatment). For the human genome RRBS determines the methylation status of 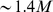 CGs [23].

We determine the span of a methyltyping method by considering regions in which that method profiles methylation. By doing so we gain an insight to the broadness of a method with respect to the regions for which it profiles methylation. In MethylSeq, methylation status is determined for a subset of the CCGG sites and in RRBS methylation status is determined for CG sites that are within fragments that have CCGG sites on both ends and which are of relative short length (up to 220bp). We therefore computationally categorized all CCGG sites of the human genome as 1/0 based on the ability to infer their methylation state with each method. All regions (bounded by CCGG sites) in which all CCGG sites received a “1” were considered as spanned by the method. When determining the span for CpG islands, the regions spanned were computed in the same manner, but considered only regions within CpG islands. In cases that the CCGG nearest to an edge of the island was determined as “1” the region between that CCGG and the edge of the island was also considered as spanned.

### Coverage of Ball MP et al. (Nat Biotech 27:361–368 (2009))

In the protocol used for this study the genome is digested with the methylation-sensitive restriction enzyme HpaII and only CCGG sites that follow certain criteria (as outlined in Ball MP et al.) are considered for their methylation status. One of the requirements is that the CCGG site be at least 40bp away from at least one of its two neighboring CCGG sites. In the human genome 19% of the CCGG sites have both of their neighboring CCGG sites at a distance smaller than 40bp, and are therefore excluded from the analysis.

### Characterization of False-Positive UCSC CpG Islands

CpG islands in the UCSC track are defined in [34] as regions with a GC content of 50% or more, a length greater than 200bp, and a greater than 0.6 ratio of observed CG dinucleotides to the expected number based on the GC content of the segment. The segments to consider are collected by scoring all dinucleotides (+17 for CG and −1 for others) and identifying maximally scoring segments. Under this model, the probability that a region from the null model (sequence which is not an unmethylted region) fulfills these requirements increases as the length of the region decreases. This statement holds for models in which the probability of observing an A/T in the null model is larger than that of observing a C/G. This is indeed the case in humans. The likelihood of false positives in the UCSC CpG island set has been noted [33], [35].

### MethylSeq Experiment

We obtained whole blood from four young adult male humans and obtained neutrophils by first isolating peripheral blood mononuclear cells by Ficoll separation, then purifying neutrophils with anti-CD16 antibodies conjugated to magnetic beads (Miltenyi); we verified that the purified samples contain 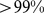 neutrophils by Wright-Giemsa staining and visual inspection by a hematologist. Genomic DNA was isolated using the DNeasy Blood & Tissue isolation kit (Qiagen), quantified using a Nanodrop spectrophotometer, and quality-controlled for purity with an Agilent Bioanalyzer. Genomic DNA (2
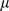
g) was digested with HpaII under conditions that make it very likely that digestion is complete (overnight with enzyme boosting), fragments 50–300bp long were isolated from an agarose gel, and single-read sequencing libraries were prepared following the manufacturer's protocol (Illumina). Libraries were sequenced on a first-generation Illumina Genome Analyzer and 32 base reads were generated. Only reads beginning with “CGG” (the sequence of the ends produced by restriction with HpaII) were retained and analyzed with MetMap.

### MetMap's Algorithm

MetMap receives as input the output of the MethylSeq experiment mapped to a reference genome, the reference genome, and the minimal and maximal lengths of the fragments sequenced, denoted by 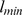 and 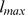. MetMap generates its graphical model (the 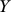, 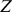 and 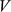 variables along with their dependency relations) from the reference genome and the values of 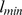 and 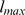. Having the graphical model's structure in place, MetMap incorporates the MethylSeq data by assigning values to all 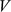 variables (all fragments that may be sequenced in the MethylSeq experiment): each 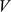 variable is assigned a score between 0 and 9, by fixing a dataset-specific “capping” value, denoted 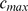 (see next section), and to each 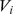, with paired-end read count 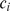, assigning 
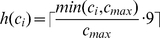
. In case of a single-end dataset a transformation approximates a paired-end dataset, and the data is scaled as if it were paired-end (Text S1). MetMap is modular, allowing for potential incorporation of methods that normalize for biases generally present in short-read sequencing technologies [36].

Several types of probability distributions annotate the dependencies between the variables of MetMap's model. The transition probabilities between each pair of adjacent variables of type 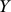 and/or 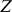 (which represent adjacent CGs) take into account the reference genome but not the MethylSeq data, and are the prior distribution over the hidden states. In case that the two adjacent variables are of type 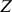, they take on a state (denoted by 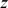) from 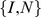. The transition probabilities are 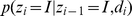, 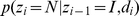, 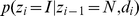 and 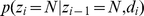, where 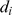 is the distance between 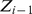 and 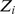. Each function determines the probability that 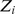 is in its island state given that 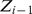 is in the given island state, that a CG is observed at 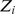 and that no CGs are observed for the distance of 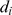. The parameters 0.00031434, 0.0257, 0.10178, 0.01298 and 0.013, respectively determine the probability of entering an island, of leaving an island, the probability of the sequence ‘CG’ occurring in an island, and out of one, and the initial probability that a site of the genome is in an island. These parameters were set using maximum likelihood estimates, calculated using chromosomes 21 and 22 of the human genome (Text S1). In cases where the successor variable of a pair of adjacent variables is of type 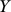, the methylation value of the state is considered. MetMap's current version assumes independence of the neighboring sites' methylation values, given the island values.

Parameters 0.2269, 0.05 and 0.7231 determine the probabilities of having an 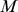, 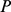 or 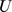 methylation value, given an unmethylated island status (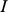). Parameters 0.8087, 0.05 and 0.1413 determine these probabilities, given an outside of unmethylated island status (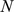) (Text S1). The transition function to any state of 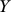 is determined as the product of the transition probability considering only island values (as specified above) and the probability of observing the methylation value of the state at hand, given its island value. The third type of probability distribution in MetMap annotates the dependencies between the 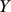 and 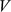 variables. Each 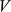 variable is dependent on the methylation values of the 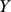 variables on the fragment it represents (Figure 3.b). Therefore, a dependency function is denoted for each 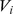 variable as 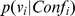, where 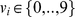 is a state of 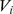 and 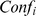 is some configuration (assignment) of the values 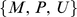 of all the restriction sites on fragment 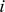. We limit the number of 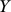 variables in the interior of such structures to at most 3 (by random choice from the interior variables), and unite methylation configurations that are equivalent with respect to the probability function, resulting in lookup tables of size at most 5×10. The parameters in the lookup table were determined using a linear program that takes into consideration the internal constraints of the probability distributions (Text S1). Artificial restriction of the number of interior variables is not common because the maximum fragment length imposed by the size selection is relatively short.

MetMap infers the posterior probabilities of its hidden states by building the junction-tree graph and using belief propagation [27]. The structure of the graph makes this computation tractable and efficient: the running time for the inference procedure is less than an hour for large chromosomes on a small sized cluster.

MetMap generates two output files. One holds for each HpaII site in the scope of the experiment a MetMap score, indicating the inferred frequency of alleles in the MethylSeq sample that are unmethylated at that site. The second file holds the coordinates and scores of the annotated SUMIs.

### Generating 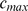


To generate a value for 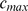, MetMap builds a histogram of the read count intensities for the subset of fragments of length 50–80bp, which do not hold internal restriction sites, and are located inside UCSC CpG islands. The fragments participating in the histogram contain a greatly reduced amount of bias (due to the lack of restriction sites in their interior) and are assumed to be mostly unmethylated (as they are in CpG islands). Under the assumption that the distribution of the histogram is close to Poisson, because the sequencing of fragments is equivalent to sampling them from the digest, we assume the variance is equal to the mean, and take 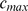 to be the value two standard deviations away from the mean of the distribution. The procedure described is carried out to avoid setting 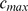 in a way which is harshly influenced by PCR amplification bias, a phenomenon that causes some sites of the genome to receive extremely high counts, regardless of the extent to which they are methylated.

### CG Sites in the Scope of the MethylSeq Experiment

MetMap outputs methylation scores only for the HpaII sites (CCGGs) that are in the scope of the MethylSeq experiment. A HpaII site is in the scope of an experiment if and only if it lies on some fragment that has HpaII sites at its ends, and is of length 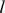 such that 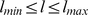, where 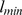 and 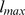 are the minimal and maximal fragment lengths for a specific MethylSeq experiment. MetMap's graphical model identifies these sites; they are all HpaII site variables (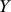 variables) that have an edge to some fragment variable (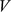 variable). Importantly, this condition does not require a site to be at an end of a fragment that satisfies the length requirements; a site may be in the interior of such a fragment.

### Annotating SUMIs

The SUMI regions annotated by MetMap are the union of two sets of regions. The first set consists of those continuous regions in which each 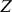 or 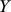 variable of the MetMap model (CG sites) received a probability of being in an unmethylated island (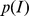) that is larger than 0.1, and in which the MethylSeq data directly supports the presence of at least two fragments. This set will include regions with relatively weak direct experimental evidence but with strong sequence evidence for being unmethylated. The second set is generated by setting a 600bp interval around each HpaII site that had a 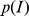 value smaller than 0.1 and a 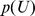 value higher than the prior probability of being unmethylated outside of an unmethylated-island (0.1663). All overlapping windows are concatenated and the regions taken are those in which at least 30% of the HpaII sites had a p(U) larger than the prior-set threshold (0.1663), and in which the MethylSeq data directly supports the presence of at least two fragments. This set includes regions with weaker sequence support for unmethylation, but with extensive evidence that they are unmethylated. Each SUMI receives a score, specifying the mean of the MetMap scores at all of the sites within the SUMI.

The SUMI lists for the four human neutrophil samples can be found at:

http://www.cs.berkeley.edu/meromit/SUMIs_Human_Neutrophil/

### Validation with Bisulfite Sequencing

DNA was treated with the MethylEasy bisulfite conversion kit (Human Genetic Signatures), PCR-amplified with locus-specific primers that recognized human target sequences, and sequenced using standard Sanger chemistry. Since all epialleles from a single specimen were sequenced in bulk in the same mixture, we estimated the ratio of unmethylated/methylated alleles at each CG in the sequence by examining the relative heights of the ‘C’ and ‘T’ traces in the sequencing output. Each CG site received a score from the set (0,0.25,0.5,0.75,1), based on the relative C/T peak height [32]. A score of 1 indicates the site is fully unmethylated, meaning that only the ‘T’ trace was observed at the C position of a given CG, while a score of 0 indicates the site is fully methylated, meaning that only the ‘C’ trace was observed at the C position of a given CG.

### “Adjusted” Bisulfite Values

We tested the extent to which our results may be affected by the representation of the bisulfite scores on a discrete five-point scale, since the true proportion of alleles that are unmethylated is a close to continuous measure. Each data point was assigned an ‘adjusted’ bisulfite score, within a tolerance window specified by the true bisulfite value of that data point. The ‘feasible ranges’ allowed for the ‘adjusted’ bisulfite scores were as follows: (0,0.15) for a 0 bisulfite score, (0.15,0.35) for a 0.25 score, (0.35,0.65) for a 0.5 score, (0.65,0.85) of a 0.75 score and (0.85,1) for a 1 score. For example, for a site with bisulfite score 0.25, read count score 0 and MetMap prediction 0.4 we would get two pairings (0.15,0) for (adjusted-bisulfite, read count score), and (0.35,0.4) for (adjusted-bisulfite, MetMap score). The score ranges were based on an assumption that assignments of “0.5” scores were the least precise. The adjustment of the bisulfite score to the read counts was done by generating a normalized read count value, in the 0–1 range, using the same “capping” value as MetMap.

### Open Chromatin ENCODE Files

One file of open chromatin was compiled from:

ftp://hgdownload.cse.ucsc.edu/goldenPath/hg18/encodeDCC/wgEncodeChromatinMap/

using the files: wgEncodeUncFAIREseqPeaksH1hesc.narrowPeak

wgEncodeUncFAIREseqPeaksNhek.narrowPeak

wgEncodeUncFAIREseqPeaksGm12878V2.narrowPeak

wgEncodeUncFAIREseqPeaksHuvec.narrowPeak

wgEncodeUncFAIREseqPeaksPanislets.narrowPeak

## Supporting Information

Figure S1 Validation of MetMap predictions by site-specific bisulfite sequencing.
(0.45 MB PDF)

Table S1 Read counts of the different samples.
(0.09 MB PDF)

Text S1 Supporting material on MetMap's algorithms and parameters.
(0.24 MB PDF)

Text S2 Supporting information on MetMap's performance and sensitivity.
(3.48 MB PDF)

Text S3 Supporting information for Figure 4.
(0.02 MB PDF)
