# Supplementary material for: MetMap Enables Genome-Scale Methyltyping for Determining Methylation States in Populations
Source: PLoS Comput Biol. 2010 Aug 19;6(8):e1000888. doi: 10.1371/journal.pcbi.1000888 (PMC2924245; doi:10.1371/journal.pcbi.1000888)
Supplement: Figure S1 — Validation of MetMap predictions by site-specific bisulfite sequencing. (0.45 MB PDF) [file pcbi.1000888.s001.pdf]

Figure S1. The different regions of the genome for which direct bisulfite sequencing was used to evaluate MetMap are presented. In each subfigure the following are shown (from top to bottom):

- Bisulfite values assigned to each CG site in the scope of sequencing, normalized to a 0-1 scale.
- Bisulfite values only for the HpaII sites (CCGG sites), normalized to a 0-1 scale (this is a subset of the previous item).
- MetMap site-specific scores.
- Read counts. For this representation, any HpaII site with read counts larger than 30 was set to 30, the "capping" value for sample 4.
- SUMI regions, along with their scores
- "bona fide" islands (denoted as the SVM-island track, because these islands are inferred with the use of a support-vector machine).
- UCSC CpG islands
- RefSeq genes

The bisulfite scores of the HpaII sites are better aligned with the MetMap scores than with the MethylSeq read counts. Moreover, the overall methylation state of the region (seen in the uppermost track) is well correlated with the presence or absence of SUMIs, and with the SUMI scores. The SUMI scores are not the mean of the MetMap scores in the validated regions because the regions validated were only a small portion of the SUMIs, which may have regions that are methylated to different extents.

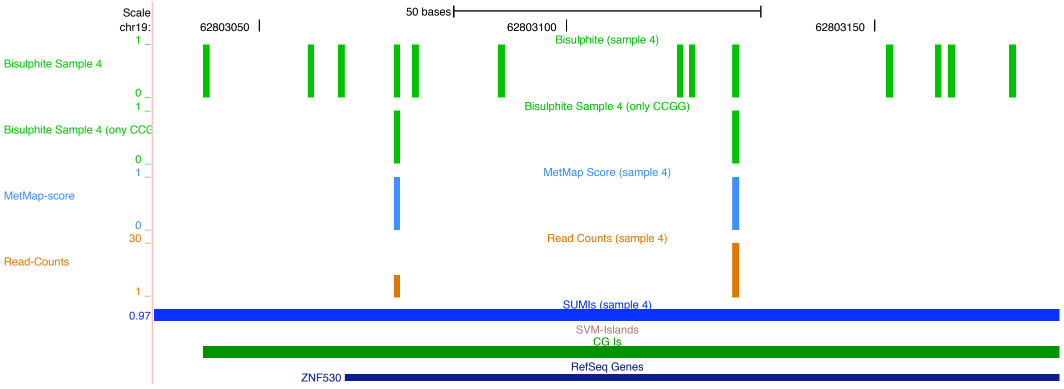

(a) chr19-62,803,034-62,803,180

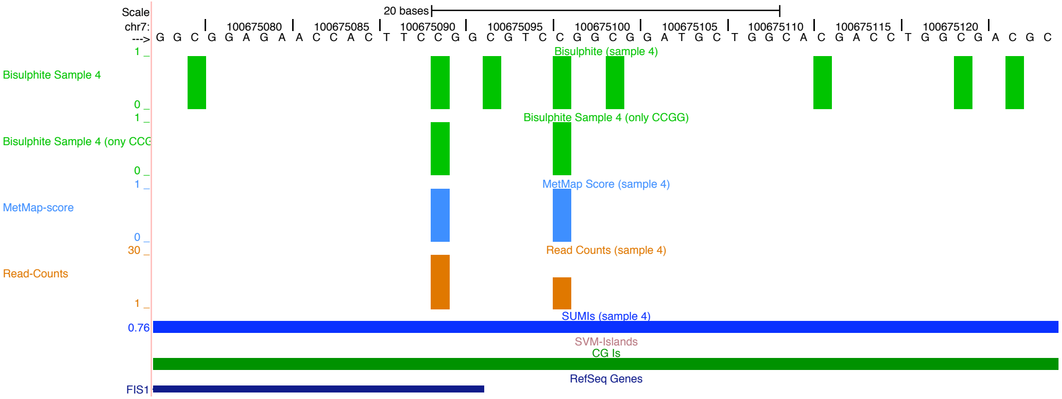

(b) chr7-100,675,073-100,675,124

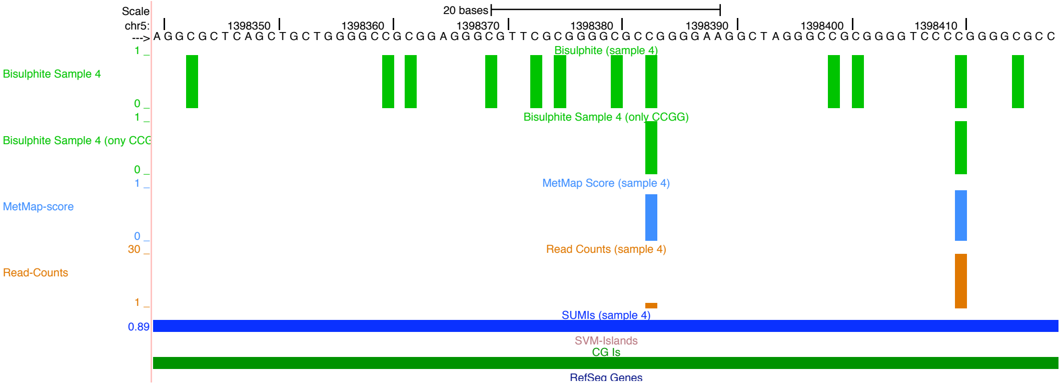

(c) chr5-1,398,340-1,398,418

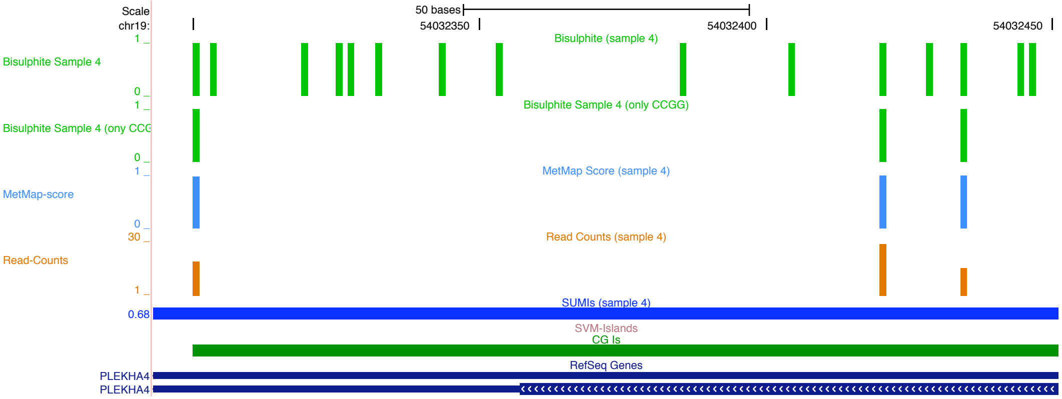

(d) chr19-54,032,294-54,032,451

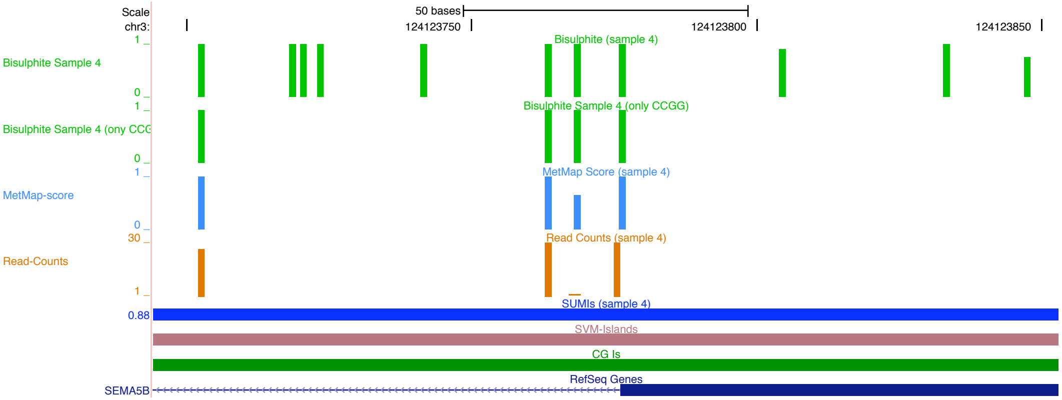

(e) chr3-124,123,695-124,123,853

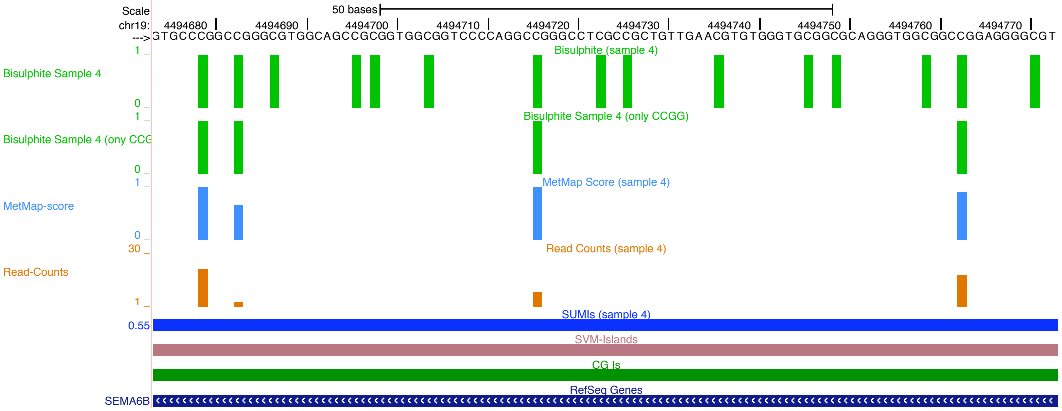

(f) chr19-4,494,674-4,494,773

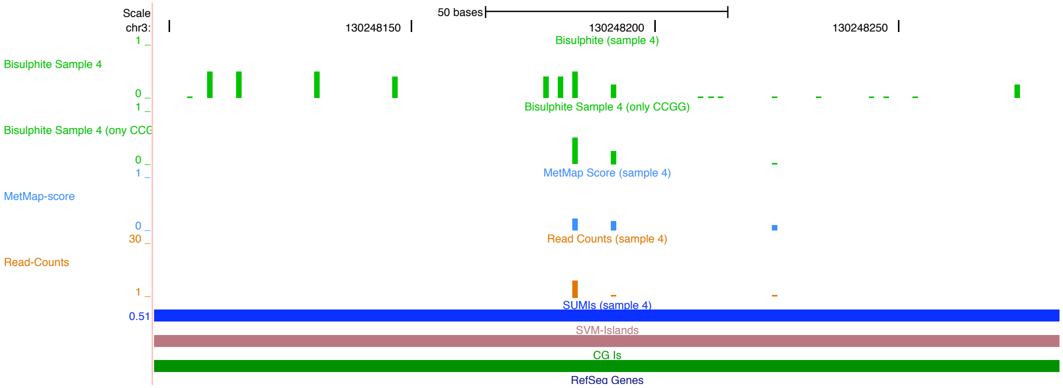

(g) chr3-130,248,098-130,248,283

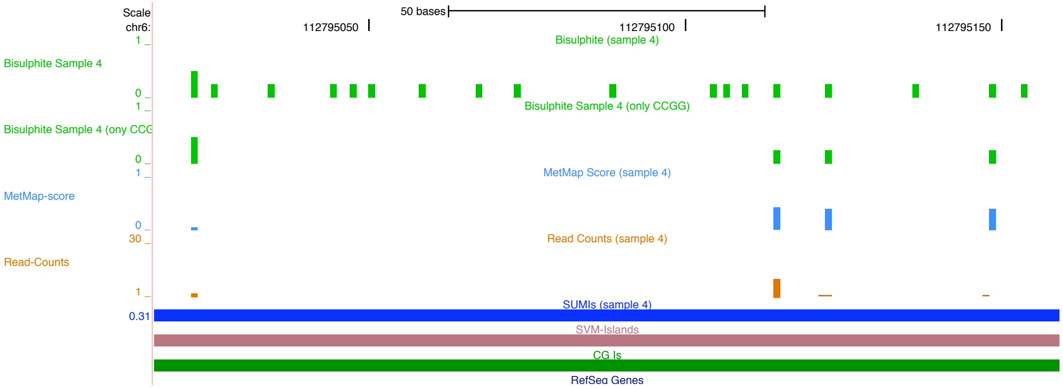

(h) chr6-112,795,017-112,795,159

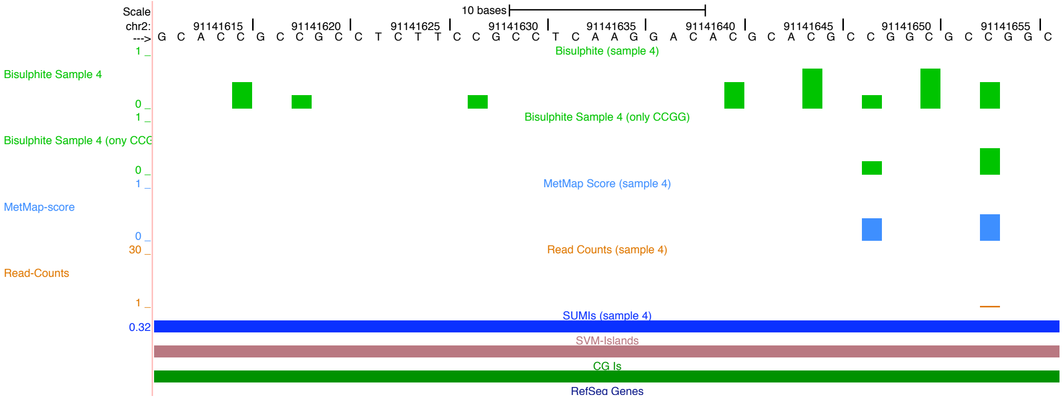

(i) chr2-91,141,611-91,141,656

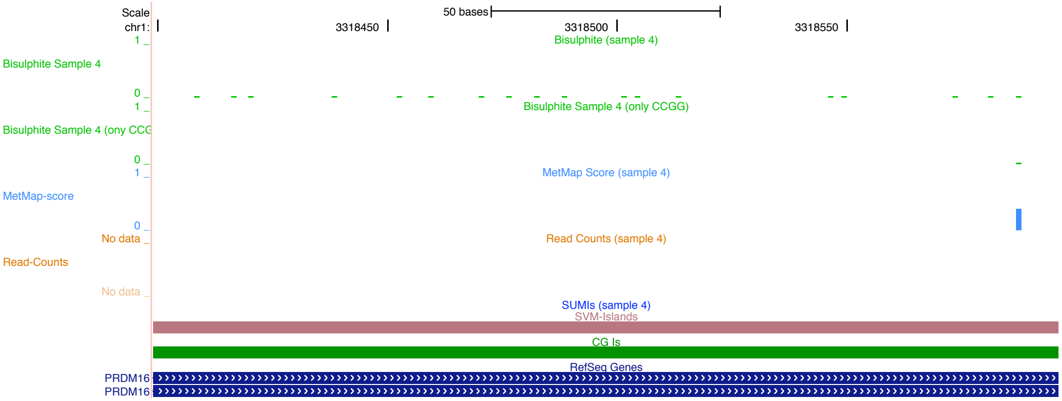

(j) chr1:3,318,400-3,318,596

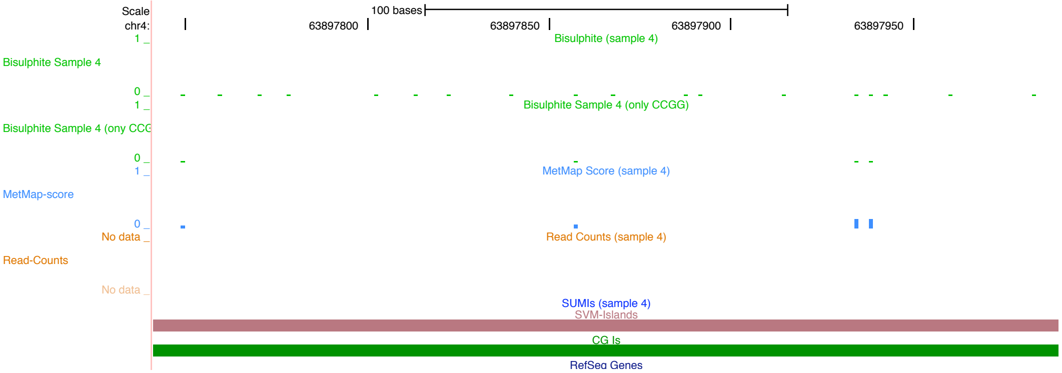

(k) chr4:63,897,742-63,897,990

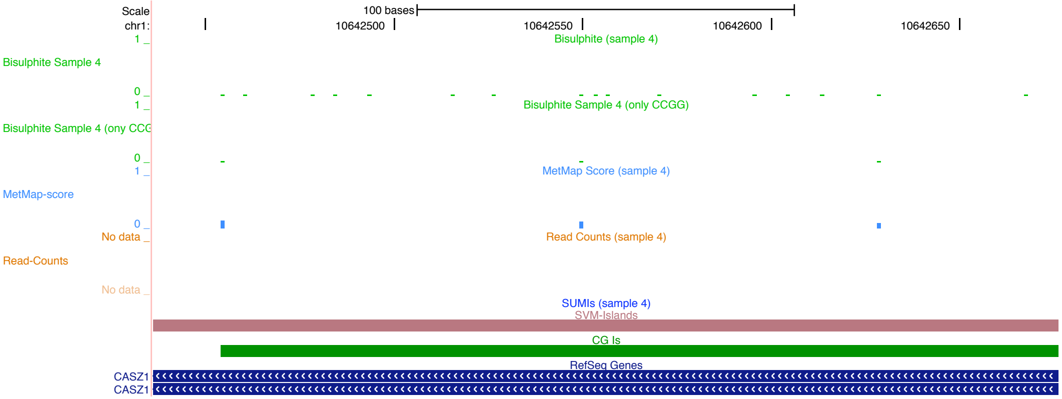

(l) chr1:10,642,437-10,642,676

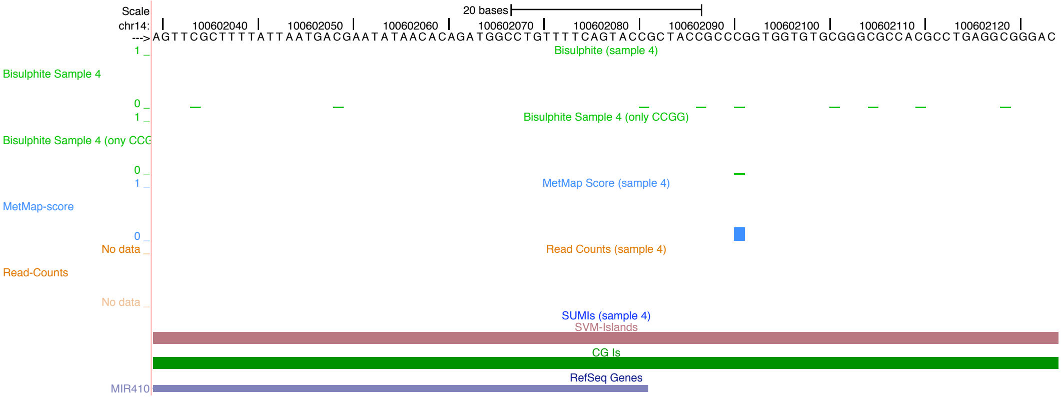

(m) chr14-100,602,030-100,602,124

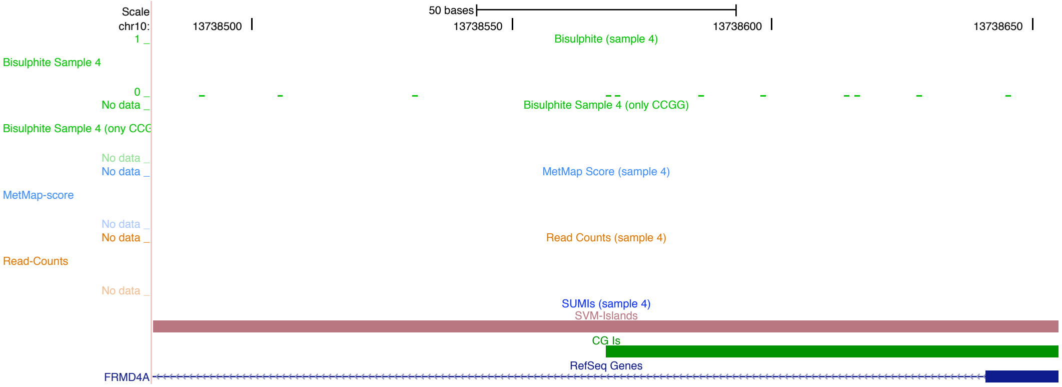

(n) chr10:13,738,482-13,738,655

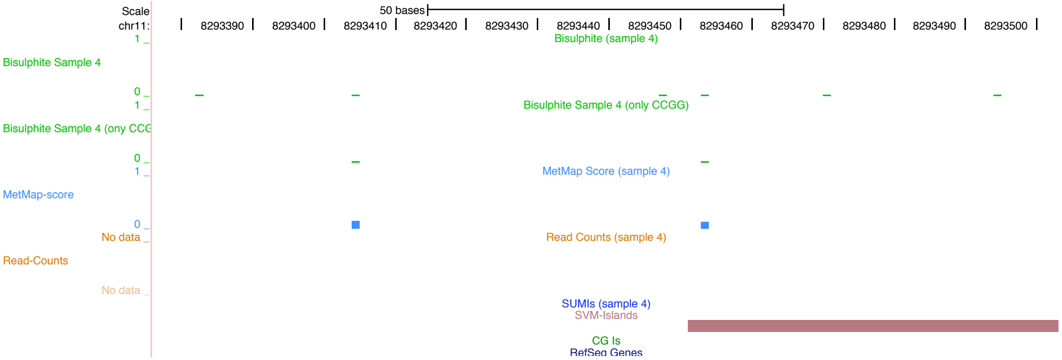

(o) chr11:8,293,377-8,293,503

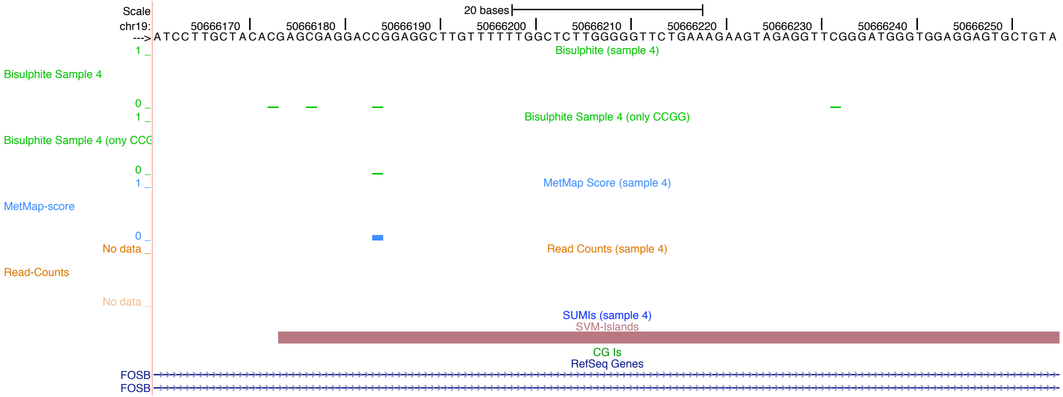

(p) chr19-50,666,161-50,666,255

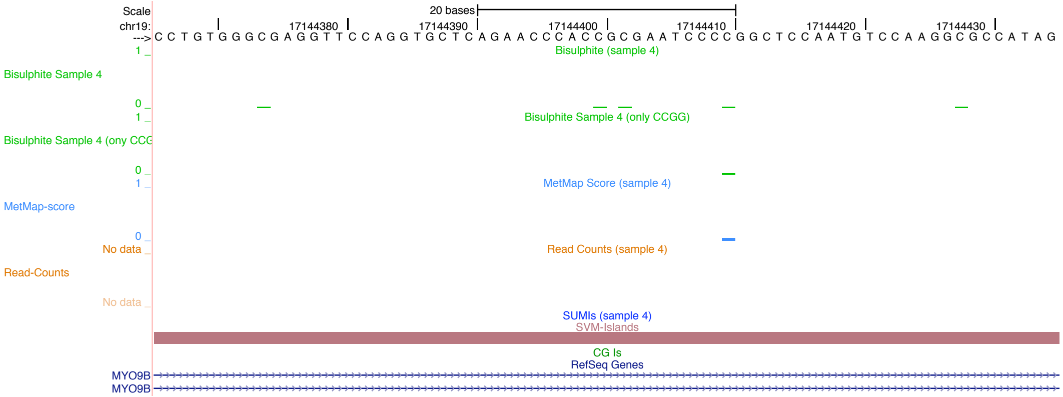

(q) chr19-17,144,366-17,144,435

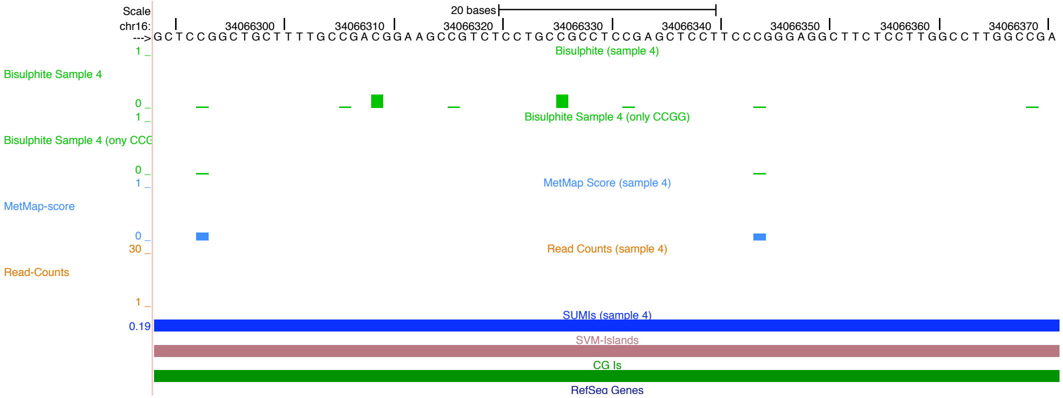

(r) chr16-34,066,289-34,066,371

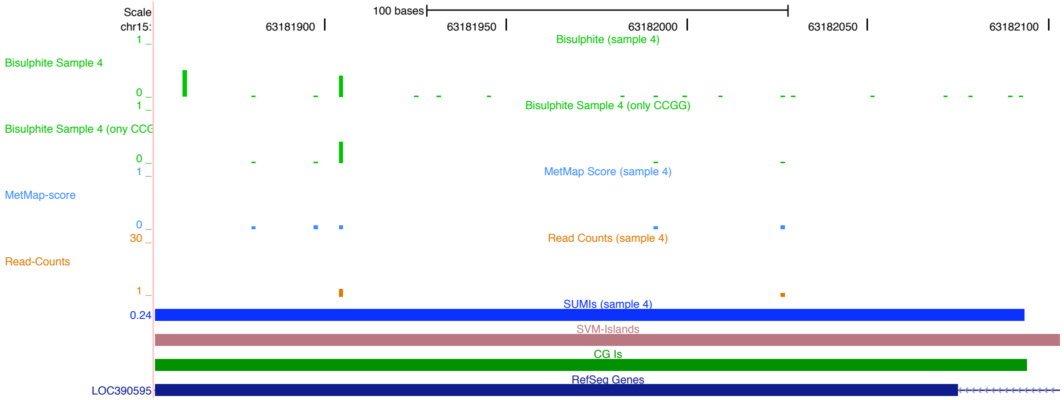

(s) chr15-63,181,854-63,182,103

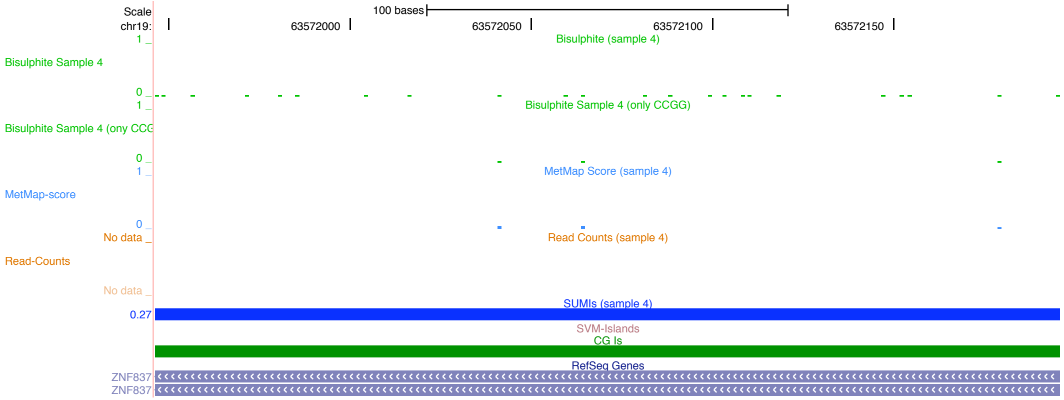

(t) chr19-63,571,947-63,572,196

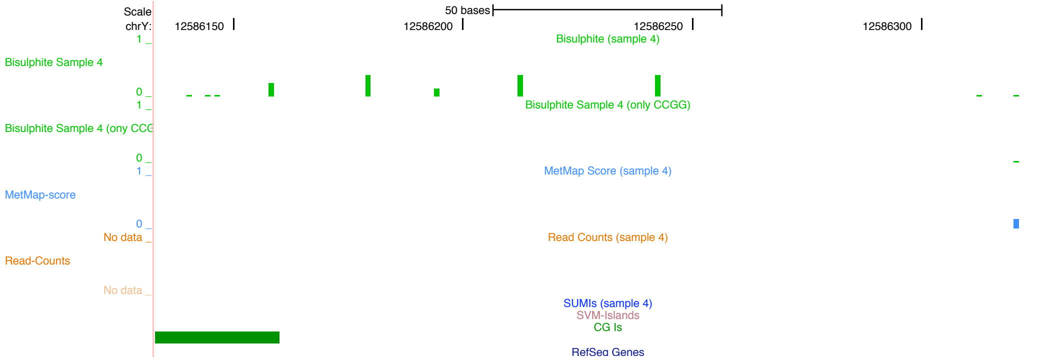

(u) chrY-12,586,134-12,586,330

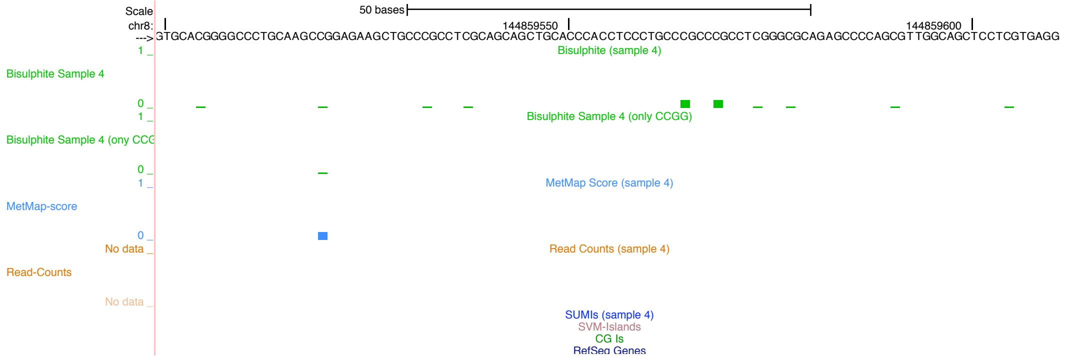

(v) chr8:144,859,500-144,859,611
