# Supplementary material for: MetMap Enables Genome-Scale Methyltyping for Determining Methylation States in Populations
Source: PLoS Comput Biol. 2010 Aug 19;6(8):e1000888. doi: 10.1371/journal.pcbi.1000888 (PMC2924245; doi:10.1371/journal.pcbi.1000888)
Supplement: Table S1 — Read counts of the different samples. (0.09 MB PDF) [file pcbi.1000888.s002.pdf]

	Sample 1	Sample 2	Sample 3	Sample 4
**Reads Sequenced**	6,227,749	6,202,253	6,233,646	5,067,711
**Alignments at HpaII sites**	4,536,673	4,602,001	4,614,571	4,465,175
**Unique alignments at HpaII sites**	4,160,629	4,221,245	4,225,504	4,066,062
