# Supplementary material for: MetMap Enables Genome-Scale Methyltyping for Determining Methylation States in Populations
Source: PLoS Comput Biol. 2010 Aug 19;6(8):e1000888. doi: 10.1371/journal.pcbi.1000888 (PMC2924245; doi:10.1371/journal.pcbi.1000888)
Supplement: Text S1 — Supporting material on MetMap's algorithms and parameters. (0.24 MB PDF) [file pcbi.1000888.s003.pdf]

Supplementary information for MetMap's algorithm

Single End Datasets

When given a single-end dataset as input MetMap performs a transformation to an approximated paired-end dataset, and proceeds in scaling of the data as if it were paired-end. Conducting a transition from a single-end dataset to the paired-end dataset representing the experiment is not a trivial manner. This is because many different paired-end datasets are consistent with data from a single-end experiment. In other words, there is no one-to-one mapping between single-end and paired-end data. Theoretically, this issue may be addressed by having an inference procedure sum over different possible paired-end assignments for the given single-end dataset; however in MetMap's case doing so would result in an infeasible inference procedure due to the large sized cliques the single-end variables would introduce. We therefore chose to use a different approach.

Given a single-end dataset, each restriction site has a read count in both the upstream and the downstream directions, indicating the number of reads generated from sequencing starting at that restriction site and proceeding to a specific direction. MetMap generates a c_{max} value using a read count intensity histogram, as described in the previous section. MetMap assumes that given a read count score at a site for a specific direction, it is most probable that most of the score originated from the shortest fragment for which there is an abundant signal at the corresponding site (the restriction site at the other end of the fragment). Dynamic scores are assigned to the different sites and different directions, starting out with each dynamic score being $\min(c_i, c_{max})$, where c_i is the raw read count. Then, the method goes through the fragments of the genome from shortest to longest. When reaching a fragment, i, it is assigned a score $v_i = min(d_b, d_e)$ where d_b and d_e are the dynamic signals of the sites at the beginning and end of the fragment, in the corresponding directions. The method then updates each dynamic signal by subtracting v_i from its dynamic score. After all assignments are through the leftover dynamic scores are distributed to the shortest fragment they are at an end of.

MetMap's Parameters and transition functions

Parameters 0.2269, 0.05 and 0.7231 determine the probabilities of having an M,P or U methylation value, given an unmethylated island status (I). Parameters 0.8087, 0.05 and 0.1413 determine these probabilities, given an outside of unmethylated island status (N). Four parameters are sufficient to define these distributions, as each distribution sums to one. The parameters for U and M were determined by the maximum likelihood estimators attained when considering the raw read counts from one experiment, annotating all sites with one or more read mapped as unmethylated. The likelihood was calculated using the reference human genome (hg18) along with the CpG island annotations of the UCSC genome browser. The value for P was set to 0.05 to encourage assignment of U and M states and to accommodate this 0.025 was subtracted from the maximum likelihood estimators for U and M.

Details of the $p(v_i|Conf)$ distribution and parameters

A dependency function is denoted for each feasible segment as $p(v_i|Conf_i)$, where $v_i \in \{0, .., 9\}$ is a state of V_i and $Conf_i$ is some configuration (assignment) of the states $\{M, P, U\}$ to all the restriction sites on fragment i. Given that there are k restriction sites on fragment i, there are 3^k different configurations possible, and therefore the size of the table of the probability distribution is $3^k \cdot 10$. The restrictions on the length of feasible fragments result in small values of k, though there are some instances k is not sufficiently

small, specifically in repeats. MetMap restricts k to be smaller or equal to 5; if the true number of restriction sites on a fragment is larger, the two sites at the end of the fragment along with three randomly chosen inner sites are considered for representing $Conf_i$ for that fragment. This decreases the amount of memory the inference process uses by a considerable amount, while not affecting the results to a large extent, as such areas tend to be rich with feasible fragments. Though there may be $3^5 \cdot 10$ values in the table of the probability distribution for a V_i variable, we have used symmetry present in the different configurations to result in a table of size $5 \cdot 10$, which represents probabilities of observing the different v_i states given any of 5 generalizations of configurations. The value for $p(v_i|Conf_i)$ is determined as $p(v_i|GenConf_i)$ where $GenConf_i$ is the generalized configuration for $Conf_i$.

The set of generalized configurations for $k > 2$ is five, as to be explained, and for the case $k = 2$ the table holds four of the generalized configuration options. The five generalized configurations are specified as: (1) Present - both end variables are assigned the state U while all inner variables are assigned an M state. (2) Not-Present - All configurations over the $\{U, M\}$ states which are different from the "Present" configuration. For all configurations of this type we do not expect to see the fragment in the experiment as it is either not cleaved at its ends, or cleaved in the middle. Also in this class are all configurations with P states such that the non-P states determine the fragment should not be present in the digest. For example, if some variables are set to P states but an end variable of the fragment is set an M state. (3) 1-P - Configurations which have one P state and may result in a "Present" configuration for a specific assignment of P to U or M. For example, a feasible fragment holding one inner restriction site which is set to a P state while both restriction sites at the ends of the fragment are set to U states. (4) 2-P - Defined in the same manner as 1-P, for the case that two P variables are present. (5) Large-P - Defined in the same manner as 1-P, for the case of 3-5 P values in the configuration. This case is treated as if 3 P values are present while the rest of the variables are of "Present" configuration. For each of these generalized configurations a probability distribution is specified for observing the 10 different states of the V variables. As each such probability sums to 1, 45 parameters are sufficient to describe the potential function table.

It is left to describe how the probability distributions are assigned for the 5 generalized configurations. The P states are treated as alternating states between M and U, spending 50% of the time in each state. Given d elements of P in a configuration, it may be turned to a "Present" configuration for at most 1 specific assignment of states $\{U, M\}$. Therefore the probability it is in a "Present" configuration at a given time is $\frac{1}{2}^d$, decreasing as d increases. As we're specifying probability distributions, given a specific general configuration, $GenConf_i$, we have $\sum_{s \in \{0,\ldots,9\}} p(s|GenConf_i) = 1$. An important factor when specifying the parameters for the different distributions is the ordering of the probability values for the different general configurations at each fixed state of V. For instance, it is important that the "Present" configuration be more probable than the 1-P configuration to result in $V_i = 9$, where the latter is more probable to result in this score than a 2-P configuration etc. The sets of constraints for the different states of the V variables united with the constraints that the values for each general configuration over the different V states sum to one, result in a linear program. Specifying the constraints we took a solution of the linear program to specify MetMap's parameters (see Table 1 and Figure 1 of this text and).

Table 1. Parameters denoting the probability of observing the different states of a V variable for each of the methylation configurations

	$v=0$	$v=1$	$v=2$	$v=3$	$v=4$	$v=5$	$v=6$	$v=7$	$v=8$	$v=9$
"Present"	0.0017	0.0126	0.0244	0.0366	0.0480	0.1047	0.1233	0.1687	0.2105	0.2691
"Not-Present"	0.4591	0.1842	0.1013	0.0770	0.0629	0.0461	0.0343	0.0228	0.0116	0.0003
1-P	0.0498	0.0619	0.0691	0.1548	0.1642	0.1642	0.1548	0.0691	0.0619	0.0498
2-P	0.0782	0.1080	0.2126	0.2126	0.1080	0.0782	0.0748	0.0523	0.0431	0.0319
Large-P	0.1470	0.3196	0.1515	0.1064	0.0854	0.0609	0.0510	0.0368	0.0259	0.0149

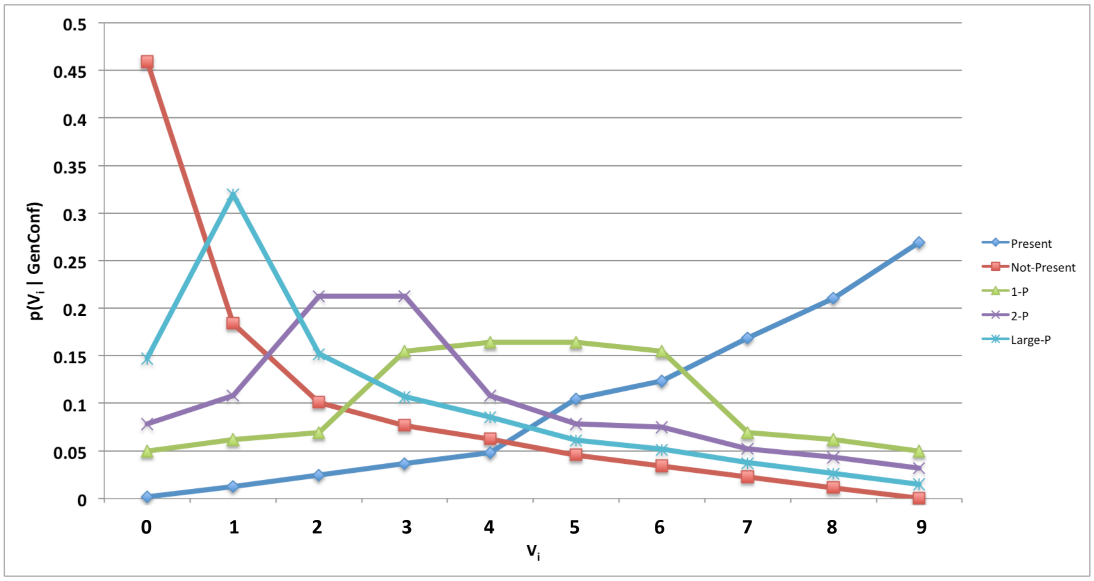

Figure 1
