# Supplementary material for: MetMap Enables Genome-Scale Methyltyping for Determining Methylation States in Populations
Source: PLoS Comput Biol. 2010 Aug 19;6(8):e1000888. doi: 10.1371/journal.pcbi.1000888 (PMC2924245; doi:10.1371/journal.pcbi.1000888)
Supplement: Text S2 — Supporting information on MetMap's performance and sensitivity. (3.48 MB PDF) [file pcbi.1000888.s004.pdf]

MetMap's inference procedure for region chr19:62799011-62807239

We illustrate MetMap's procedure in a region with a small number of CCGG sites. The region chosen has 11 CCGG hidden variables, and there are no CCGG sites 300bp upstream of the first variable or 300bp downstream of the second variable. In Figure 1 of this text we specify the number of read-counts, of normalized read counts, of paired-end read counts deduced from single ends, of the structure of MetMap for that region, and of the posterior probabilities inferred for each site.
We note that the fragments 2-...-5 and 2-...-8 were probably not present in the solution but are still assigned low read counts due to MetMap's procedure for annotating paired-end counts from single-end counts. This example illustrates the information present in the data involving locations to which no reads have been mapped, which MetMap takes into consideration in its inference procedure. For example, by examining part (a) of the figure, we could notice that sites 2 and 6 are definitely unmethylated. We could then notice that if site 5 were methylated, we would get some reads mapping to site 6 in the upstream direction (whether they would originate from sites 4 3 or 2, the latter which we know to be unmethylated). Therefore, site 5 must also be unmethylated. Under the same reasoning we can reach the conclusion that site 4 is unmethylated (otherwise we would have more reads mapped to site 5) and that site 3 is unmethylated (otherwise we would get reads at the upstream direction of site 4). The same goes for site 8: even though this site got a lower read count, the absence of reads in the upstream direction of site 9 indicate site 8 is highly unmethylated. The inference at site 8 is an example where MetMap accounts not only for size selection, but also for the randomness present when sampling fragments to sequence from a digest.

MetMap's Sensitivity

We chose to demonstrate MetMap's sensitivity in two different ways:

1) We first examined a case where different human samples (sample 1 and sample 4) had different read-counts for the same region of the genome, and show that MetMap's predictions are sensitive to this difference. Table 1 shows for each site in the region the read count and MetMap inference for each of the two samples.

2) We tested MetMap's sensitivity by artificially changing the read-counts at a region and evaluating MetMap's inferences at that region given the different input. For region chr19:4494679-4494763 that was predicted by MetMap as unmethylated for the actual sample data, and validated using bisulfite sequencing, we evaluated MetMap's inferences for the case that region was methylated. For the simulated case of the region being methylated (with no read-counts) the MetMap scores dropped considerably (see Table 2).

   We believe that the reason the MetMap scores for the methylated case are around 0.1 (and not closer to 0) is the abundance of CG sites at that area, which affects the prior distribution, pushing towards the possibility of being unmethylated. In future versions of MetMap we will approach this issue, and attempt to improve on the sensitivity of MetMap by allowing for extended flexibility in the prior distribution.

Table 1: **Different Read Counts in samples result in different MetMap predictions**

	Sample 1		Sample 4	
Coordinate	Read Counts	MetMap Score	Read Counts	MetMap Score
chr16:66244444	5	0.069005	15	0.6234
chr16:66244465	2	0.128735	12	0.71821
chr16:66244537	1	0.21895	13	0.722445
chr16:66244541	2	0.24566	7	0.70159
chr16:66244599	5	0.592835	12	0.71721

The area of chr16:66244444-66244599 (156 bps) has consistent different read counts across the region for two human samples. This difference is reflected in MetMap's output for this region in each of the different samples.

Table 2: **MetMap scores for validated region, for different read-count inputs**

Coordinate	Unmethylated (Original Input)	Methylated (Simulated Input)
chr19:4494679	0.999315	0.10772
chr19:4494683	0.76112	0.108185
chr19:4494716	0.97853	0.130815
chr19:4494763	0.9005	0.10113

Sites validated as unmethylated with bisulfite sequencing receive a significantly lower MetMap score when MetMap is given no read-counts for sites in the region, simulating it being methylated.

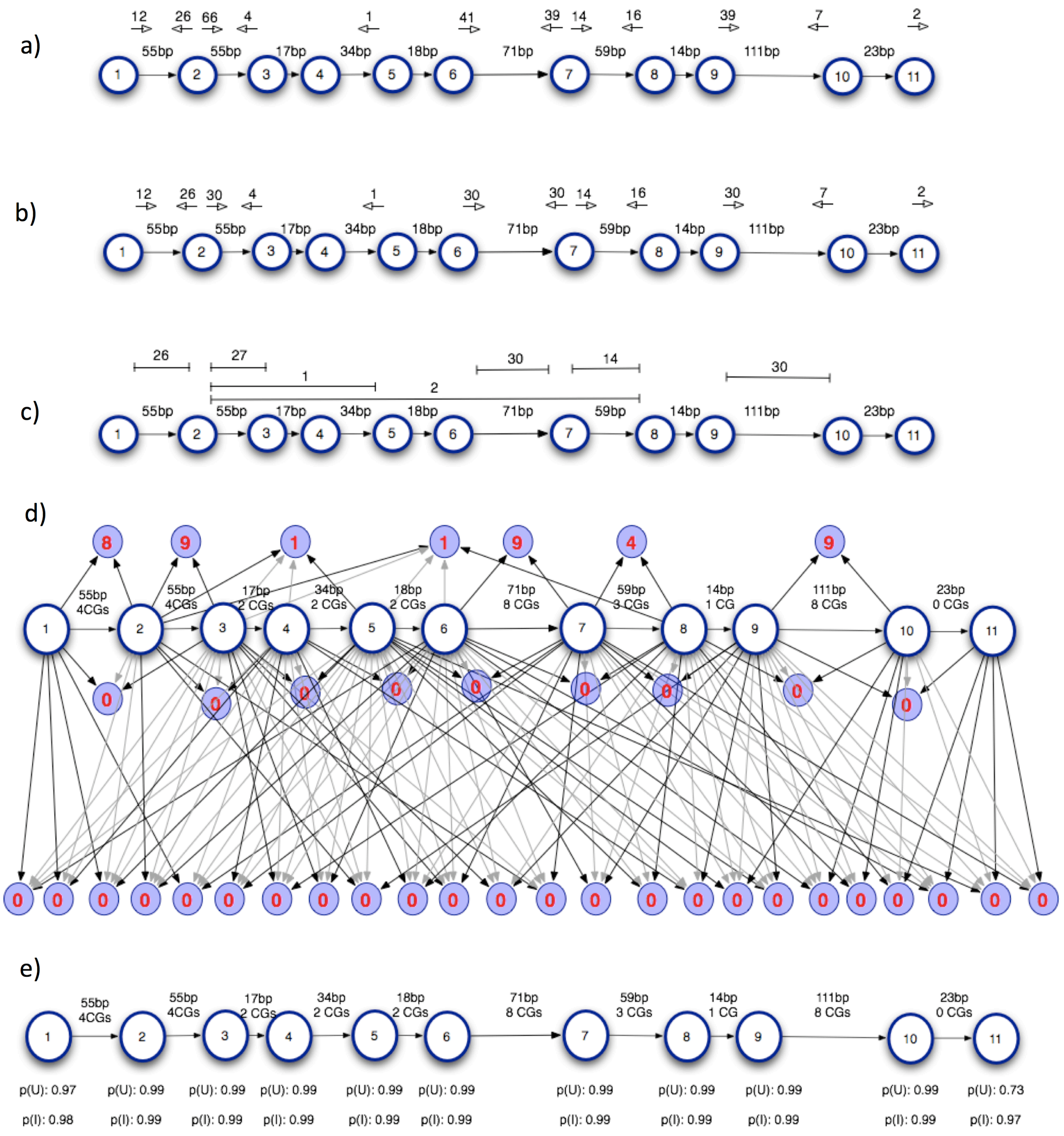

Figure 1: **An example of MetMap's inference procedure for region chr19:62799011-62807239 in sample 4.** For the region at hand MetMap's graphical model has 11 hidden variables for the CCGG sites and 34 hidden variables for other CG sites (not shown). MetMap first takes in the raw read counts (a), normalizes them by the capping value chosen for sample 4 (b), and deduces paired-end counts (c). MetMap then builds its graphical model (d) and runs the junction tree algorithm, assigning posterior probabilities for the CCGG variables of being Unmethylated ($p(U)$) and of being part of an Unmethylated Island ($p(I)$).
