# Supplementary material for: MetMap Enables Genome-Scale Methyltyping for Determining Methylation States in Populations
Source: PLoS Comput Biol. 2010 Aug 19;6(8):e1000888. doi: 10.1371/journal.pcbi.1000888 (PMC2924245; doi:10.1371/journal.pcbi.1000888)
Supplement: Text S3 — Supporting information for Figure 4. (0.02 MB PDF) [file pcbi.1000888.s005.pdf]

Further discussion of Figure 4

These plots illustrate the extent of consistency among samples. In all comparisons some sites are determined as fully unmethylated in one sample and unmethylated to some extent in the second sample. These can be seen as lines from the top right corner towards the bottom right corner, and from the top right corner towards the top left corner. These are sites that differ in methylation between the two samples, and the plots show that when a site differs in its methylation state between two samples, in the large majority of the cases the site is completely unmethylated in one of the two samples. At the lower right and upper left corners of the plots there are concentrations of sites that are determined to be highly, but not completely, unmethylated in one of the samples. We hypothesized that these concentrations occur because in those sites the prior (which is based on the genomic region in which the sites are located) has influenced the MetMap scores to be slightly lower than their true biological states. To test this hypothesis, we took the sites within such a concentration and determined the proportion that are part of the UCSC CpG island set, and then compared this to the proportion in other subsets of sites. For our test we used the comparison between samples 3 and 4, and ran our test on the sites at the lower right corner of the plot. Specifically, we used sites that received a MetMap score for sample 3 that was between 0.1 and 0.4, and a MetMap score for sample 4 that was between 0.85 and 0.96. Of all 202,284 sites in the plot that compares samples 3 and 4, 80% are inside UCSC CpG islands. Of the sites that received a MetMap score lower than 0.4 for sample 3 (the sites in the lower half of the plot), 66.4% are inside UCSC CpG islands. Of sites with a MetMap score between 0.1 and 0.4 for sample 3, and a MetMap score higher than 0.96 for sample 4, 64.3% are within UCSC CpG islands. Finally, of sites with a MetMap score between 0.1 and 0.4 for sample 3, and a MetMap score between 0.85 and 0.96 for sample 4 (i.e. the sites that are part of the concentration), 31.2% are in UCSC CpG islands. This outcome supports our hypothesis, because the sites present in the concentration at the lower right corner are much less frequently part of a UCSC CpG island, and this characteristic affects the prior. These findings show that MetMap's prior distribution does have an effect on the inferences of MetMap, pulling scores down at times, but the extent to which the current prior distribution has changed the inferences for these sites is not large, and easily allows these sites to be detected as highly unmethylated.
